# An LC-MS/MS-based platform for the quantification of multiple amyloid beta peptides in surrogate cerebrospinal fluid

**DOI:** 10.1016/j.jmsacl.2024.01.002

**Published:** 2024-01-22

**Authors:** Merve Oztug, Bilgin Vatansever, Gonca Altin, Muslum Akgoz, Suleyman Z. Can

**Affiliations:** TUBITAK National Metrology Institute (TUBITAK UME), Kocaeli, Turkey

**Keywords:** Amyloid beta peptides, Cerebrospinal fluid, Quantification, Solid-phase extraction

## Abstract

**Introduction:**

The accurate quantification of amyloid beta (Aβ) peptides in cerebrospinal fluid (CSF) is crucial for Alzheimer's disease (AD) research, particularly in terms of preclinical and biomarker studies. Traditional methods, such as the enzyme-linked immunosorbent assay (ELISA), have limitations. These include high costs, labor intensity, lengthy processes, and the possibility of cross-reactivity.

**Objectives:**

The primary objectives of this research were twofold: to comprehensively characterize Aβ peptides and to develop a reliable and accurate method for the simultaneous quantification of Aβ 1–40 and Aβ 1–42 peptides in surrogate CSF that is traceable to the International System of Units (SI).

**Methods:**

We developed a novel method that combined solid phase extraction (SPE) with isotope dilution liquid chromatography/tandem mass spectrometry (ID-LC/MSMS). SPE was employed to efficiently eliminate matrix interferences, while [15N] Aβ1-40 and [15N] Aβ1-42 served as internal standards to improve accuracy. In addition, we introduced Peptide Impurity Corrected Amino Acid Analysis (PICAA) to ensure traceability to the SI and reliable quantification of Aβ peptides.

**Results:**

The developed platform demonstrated a linear calibration range of 300–20000 pg/ml for both Aβ1-42 and Aβ1-40 peptides, accompanied by strong correlation coefficients greater than 0.995. Quality Control (QC) samples demonstrated an accuracy of at least 90.0 %.

**Conclusion:**

The enhanced specificity and flexibility of the developed platform potentially have implications for Alzheimer's disease diagnosis and future investigations of novel Aβ peptide biomarkers.

## Highlights

• An LC-MS/MS method was developed to accurately quantify Aβ peptides in CSF.
• Uncertainty was evaluated in the method for precise quantification of Aβ peptides.
• The PICAA method is utilized in reference material value assignment.

## 1 Introduction

Alzheimer's disease (AD) is a neurodegenerative disorder characterized by cognitive decline and memory loss, associated with the accumulation of amyloid beta (Aβ) peptides in the brain [1]. Aβ peptides, derived from the amyloid precursor protein (APP), play a crucial role in the formation of neurofibrillary tangles and senile plaques, particularly in the hippocampus [2]. The peptides Aβ1-40 and Aβ1-42 have been extensively studied due to their involvement in AD pathology [3], [4]. Quantifying Aβ peptides in cerebrospinal fluid (CSF) serves as a vital tool for diagnosing and researching AD. Aβ40 is more abundant in cerebrospinal fluid (CSF); its reported levels are about 2–20 ng/mL, compared to the 0.2–1 ng/mL of Aβ42, which can decrease in AD patients by up to 50 % [5], [6], [7]. Often, the Aβ40 to Aβ42 ratio is measured because the Aβ42 concentration exhibits significant changes due to aggregation in AD, while Aβ40 remains relatively stable [4]. Quantitative measurements of Aβ peptides in CSF are most commonly conducted using enzyme-linked immunosorbent assays (ELISA), which use multiple antibodies with high affinity for Aβ peptides [5], [6]. However, methods like ELISA have limitations, including cost, labor intensity, time consumption, and cross-reactivity [3], [8]. Consequently, there is growing interest in developing alternative methods that offer improved accuracy, specificity, and efficiency in quantifying Aβ peptides. In addition to ELISA, positron emission tomography (PET) scans using 18F-fluorodeoxyglucose are used to visualize amyloid deposits in the brain, providing a visual correlation with senile plaques observed in autopsies [7]. This imaging technique has contributed to our understanding of the spatial distribution and accumulation of Aβ peptides in the brain.

In this context, this study aimed to develop alternative methods to address the limitations of traditional quantification techniques. The focus was on improving accuracy, specificity, and efficiency in Aβ quantification. Ensuring traceability in clinical analysis is essential for reliable and comparable results across different laboratories [9]. Traceability is established by maintaining an unbroken and documented chain of calibrations, where each calibration contributes to the measurement uncertainty. This ensures that results can be traced back to preceding calibrations [9]. While primary reference measurement procedures based on the International System (SI) units are ideal, the use of certified reference materials and reference measurements is common in practice [10], [11], [12].

In this context, this study aimed to fulfill two primary objectives: firstly, to comprehensively characterize Aβ peptides using the Peptide Impurity Corrected Amino Acid Analysis (PICAA) approach, and secondly, to simultaneously quantify Aβ 1–40 and Aβ 1–42 peptides in surrogate CSF through the creation and validation of a solid-phase extraction (SPE) and isotope dilution liquid chromatography/tandem mass spectrometry (ID-LC/MSMS) platform. Our goal was to establish traceability in the quantification of Aβ peptides, adhering strictly to calibration procedures and metrological standards. We designed the SPE sample preparation protocol to eliminate matrix interferences and enhance specificity with [15N] Aβ1-40 and [15N] Aβ1-42 serving as internal standards for accurate quantification. We employed mass spectrometry, known for its high sensitivity and selectivity, for Aβ peptide quantification [9], [13], [14], [15], [16]. To ensure traceability and enhance the accuracy of measurements, we incorporated the PICAA method [17], [18], [19]. This method is crucial for the accurate determination of the mass fraction of the calibration materials, consequently ensuring reliable measurements. We applied and validated this approach for both amyloid beta 1–40 and 1–42 peptides to establish the value of the pure calibrator materials used in our quantification process. Incorporating the PICAA method in our approach contributes to achieving traceability and improving the overall accuracy of our quantification method for Aβ peptides. Furthermore, the platform shows potential for the quantification of additional Aβ peptides, which could contribute to the identification of novel AD-related biomarkers [3]. The availability of a reliable quantification method contributes to our understanding of AD pathology and could impact the development of novel biomarkers and advancements in AD research.

## 2 Materials and methods

### 2.1 Chemicals and materials

Nitrogen-15 isotopically labeled human Aβ peptides [15N] Aβ1–40 and [15N] Aβ1–42, as well as synthetic human Aβ peptides Aβ1–40 and Aβ1–42, were obtained from rPeptide (Athens, GA, USA). Milli-Q lab water system (Millipore, Billerica, MA, USA) was used to obtain water for the mobile phase and sample preparation. Acetonitrile, ammonium hydroxide, and *ortho*-phosphoric acid were purchased from Merck (Darmstadt, Germany). Potassium dihydrogen phosphate and sodium chloride were purchased from Applichem (Darmstadt, Germany). Methanol, potassium chloride, and guanidine HCl were purchased from VWR (Pennsylvania, USA). Sodium hydroxide and sodium bicarbonate were sourced from Sigma (Missouri, USA). Magnesium sulphate and calcium chloride were purchased from Fluka (Germany). Pooled human CSF was purchased from Innovative Research (USA). Artificial CSF was prepared from 122 mM NaCl, 3 mM KCl, 0.4 mM KH2PO4, 1.3 mM CaCl2, 25 mM NaHCO3, 1.2 mM MgSO4, and 0.4 % bovine serum albumin [13], [20].

### 2.2 PICAA analysis

The reagents used in the study included Certified Reference Materials (CRMs) from National Metrology Institute of Japan (NIMJ) for the natural amino acid standards or calibration standards: L-alanine (Ala, CRM 6011-a, 99.9 ± 0.2 %), L-phenylalanine (Phe, CRM 6014-a, 99.9 ± 0.2 %), and L-valine (Val, CRM 6015-a, 99.8 ± 0.2 %). For the ID-LC/MSMS method, internal standards of labeled amino acids were obtained from Cambridge Isotope Laboratories. These internal standards included: Ala (13C3, 99 %; 15 N, 99 %), Phe (13C9, 99 %; 15 N, 99 %), and Val (13C5, 99 %; 15 N, 99 %).

#### 2.2.1 Amino acid analysis

The article by Oztug et al., published in 2023 [21], provides detailed information on the amino acid analysis procedure. Briefly, the natural amino acid mixture was prepared to match the concentrations found in amyloid peptides. Fresh preparations were made for each hydrolysis reaction. Amino acid calibration standards were prepared at concentrations corresponding to 12.5 µM, 25 µM, 50.0 µM, 100 µM, 200 µM, and 400 µM beta amyloid, with each solution being prepared accordingly. A six-point calibration curve was generated by combining the natural and labelled amino acid mixtures. The hydrolysis of the amyloid peptides was conducted under inert atmospheric conditions at 130 °C for 24 h using hydrolysis tubes containing 200 µL of 6 M constant boiling HCl and 1 % phenol. The samples were then evaporated under vacuum until dry and reconstituted with 200 µL of 20 mM HCl. The amino acids were derivatized using propyl chloroformate, followed by LC-MS analysis as described in Oztug et al., 2023 [21]. For a more comprehensive understanding and exact methodology, referring to the original article is recommended.

#### 2.2.2 Intact peptide analysis

Experiments were performed using an UltiMate™ 3000 UHPLC system (Thermo Scientific), and coupled with an OrbiTrap-Q Exactive High Resolution Mass Spectrometer (Thermo Scientific), and an Aeris Peptide C18 column (250 x 2 mm i.d., 5 µm particle size). The mobile phase comprised two components. Mobile phase A was a mixture of 95 % water (H2O) and 5 % acetonitrile, with 0.1 % formic acid (FA), while mobile phase B consisted of 5 % water in acetonitrile, also with 0.1 % FA. The flow rate throughout the analysis was maintained at 400 µL/min. The gradient began with an initial composition of 5 % solvent B and gradually increased to 35 % solvent B over 25 min. After this gradient, wash and equilibration steps were performed. During the entire analysis, the column temperature was held at 40 ℃. The injection volume was 5 uL. Each sample had a final analyte concentration of 0.2 mg/mL, dissolved in a solution comprised of 95 % water. The analysis was conducted over three days, with three replicates performed each day. Each replicate consisted of ten technical replicates of each sample. Quantification was achieved through external calibration using Aβ peptides as the calibrant. The identification of peptide-related impurities was conducted using MSMS analysis.

The assignment of the amyloid value was determined through PICAA analysis, utilizing the equation (1) provided below [17], [19], [22], [23];

(1) $$x_{p}=\left(\frac{M_{r}\left(P\right)}{Z_{1}}\right)\left[\frac{n_{AA}}{m_{m}}-\sum Y_{{IMP}_{i}}\frac{X_{{IMP}_{i}}}{M_{r}\left({IMP}_{i}\right)}\right]$$

Where,

$x_{p}$ is the mass fraction of amyloid peptide in the material;

$m_{m}$ is the mass of the material analyzed;

$M_{r}\left(P\right)$ is the relative molecular mass of amyloid peptide;

$Z_{1}$ is the number of molecules of the amino acid of interest per amyloid molecule;

$n_{AA}$ is the amount of substance of the amino acid of interest measured in the material;

$Y_{{IMP}_{i}}$ is the number of molecules of the amino acid of interest per peptide impurity molecule (IMP_i_);

$X_{{IMP}_{i}}$ is the mass fraction of the peptide impurity (IMP_i_);

$M_{r}\left({IMP}_{i}\right)$ is the relative molecular mass of the peptide impurity (IMP_i_);

### 2.3 Preparation of standard solutions, calibration curves & quality control samples

Stock solutions for the individual peptides, Aβ1-40 and Aβ1-42, were purchased as lyophilized white powder with a peptide purity of over 95 %. The stock solutions were reconstituted with DMSO to achieve a final concentration of 1.00 mg/ml. Aliquots of 20 µL were prepared and stored at − 20 °C in polypropylene vials (Low Binding tubes, Eppendorf, Germany). On the day of usage, calibration and Quality Control (QC) samples were prepared gravimetrically. Calibration solutions were made by diluting stock solutions with water/acetonitrile/NH_4_OH (49:50:1) to obtain different concentrations of Aβ1-40 and Aβ1-42 standards, which included 7.5, 15, 31.125, 62.5, 125, 250 ng/ml. QC samples were prepared in a similar manner at concentrations of 25, 75, and 150 ng/ml. For the creation of working check solutions, the Aβ1-40 and Aβ1-42 standards were spiked with an equal volume of labeled internal standards ([15N] Aβ1-40 and [15N] Aβ1-42) at a concentration of 75 ng/mL each. Matrix solutions were prepared by adding 20 μL of standard and 20 μL of labeled standard to 180 μL of surrogate CSF containing 4 mg/ml bovine serum albumin (BSA).

The final concentrations of the working standard solutions were 3.5, 7, 15, 30, 60, and 115 ng/mL. The working QC solutions had concentrations of 15, 30, and 75 ng/ml. Prior to LC/MSMS analysis, the spiked solutions were purified via the SPE method.

### 2.4 SPE extraction of Aβ peptides from surrogate matrix

SPE was performed using Oasis MCX Elution, 1 cc cartridges (Waters), with some modifications, as described in the Oasis cartridge manual. The extraction process involved the use of the Agilent PPM 48 positive pressure manifold. Prior to loading the prepared samples, the SPE cartridges were washed with 600 μL of methanol and equilibrated with 600 μL of 4 % aqueous phosphoric acid. The cartridges were then washed twice with 300 μL of 4 % phosphoric acid. An equal volume of 5 mol/L guanidine hydrochloride was added to the surrogate matrix spiked with internal standards. The resulting mixtures were vortex-mixed at 1000 rpm for 20 min, followed by the addition of an equal volume of 4 % aqueous phosphoric acid (resulting in a total volume of 1800 μL). The analytes were subsequently eluted twice with 150 μL of elution buffer (comprising acetonitrile, water, and 2.5 % NH_4_OH in a 75:15:10 ratio). The eluates were collected in 1.5 mL low-binding tubes and dried using vacuum centrifugation. Before analysis, the dried samples were reconstituted in 50 μL of aqueous solution containing 20 % acetonitrile and 1 % NH_4_OH. The samples were vortex-mixed for 20 s, briefly centrifuged to concentrate the volume at the bottom of the vial, and finally transferred to 0.3-mL microvials for further analysis.

### 2.5 LC-MSMS analysis

LC analysis was performed using a Dionex Ultra Performance Liquid Chromatography (UPLC) system (Thermo Scientific, Bremen, Germany) equipped with an integrated high-pressure solvent delivery system and an autosampler. The autosampler featured a stainless steel needle operating in the partial loop needle overfill mode, with a loop size of 20 µL. For both weak and strong washes, the autosampler utilized a wash solvent composed of water, acetonitrile, and NH4OH in a ratio of 40:50:10 (v/v/v). The chromatographic separation was carried out on a Phenomenex Jupiter 5u column with dimensions of 150x2.0 mm. The mobile phase consisted of two components: Component A was prepared with 0.075 % NH_4_OH and 5 % acetonitrile in water, while Component B consisted of 0.075 % NH_4_OH and 95 % acetonitrile in water. The LC gradient program was as follows: At the beginning of the run (0 min), the mobile phase comprised 90 % A and 10 % B, with a flow rate of 200 µL/min. Over a span of 10 min, the gradient transitioned to 45 % B. Subsequently, there was a 4-minute wash phase with the mobile phase consisting of 100 % B, followed by a 3-minute equilibration period with 10 % B. Throughout the analysis, the flow rate of the mobile phase was consistently maintained at 0.20 mL/min, and the column temperature was set at 50 °C. Each sample was injected with a volume of 30 µL.

Electrospray MS-MS analysis was conducted using a Thermo Scientific Orbitrap Q-Exactive MS instrument (Thermo Scientific, Bremen, Germany), operated in positive ion mode. The instrument was optimized for each individual peptide through flow injection. The settings for the ESI (Electrospray Ionization) source spray were as follows: a source voltage of 6 kV, sheath gas at 50 arb, auxiliary gas at 6 arb, sweep gas at 5 arb, a capillary temperature set to 350 °C, an S lens RF level at 61, and an auxiliary gas heater temperature at 190 °C. Parallel Reaction Monitoring (PRM) transitions were used, and the specific transitions can be found in Table 1. The properties of both the MS and MSMS analyses were as follows: The full MS analysis was conducted with a resolution of 70,000. The Automatic Gain Control (AGC) target was set to 3e6, and the maximum injection time (IT) was set to 200 ms. The scan range for the full MS scan was set from 200 to 2000 *m*/*z*. For the data-dependent MS2 (dd-MS2) or data-dependent SIM (DD-SIM) analysis, a resolution of 35,000 was used. The AGC target was set to 1e6, and the maximum injection time was 200 ms. The instrument performed 5 loop counts, selecting the top 5 ions for fragmentation based on their intensity. The isolation window was set to 2.0 *m*/*z*, and the collision energy (CE) for fragmentation was set to 15 or 16. A six-point linear calibration curve was employed for quantification.

**Table 1:** Ions/PRM transitions monitored:

**Peptide name**	**Precursor ion**	**Product ion 4+**	**Product ion ID**	**Collision energy (NCE)**
**Aβ_1-40_**	1083.29	1054.02	b_39_^+4^ b_39_^+4^ b_40_^+4^ b_40_^+4^	15
**[^15^N]Aβ_1-40_**	1096.50	1066.73		16
**Aβ_1-42_**	1129.57	1078.79		15
**[^15^N]Aβ_1-42_**	1143.28	1091.99		16

## 3 Results and discussion

### 3.1 PICAA analysis

**Fig. 1:**
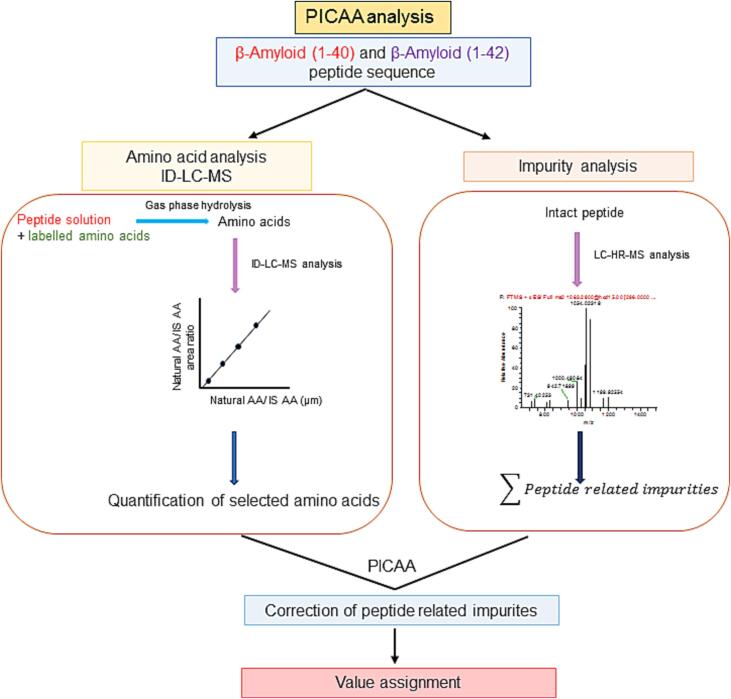
PICAA strategy for preparation of the β-Amyloid 1–40 and 1–42 peptides primary standard.

Peptides play a vital role as biomarkers and in pharmaceuticals, making the accurate purity characterization of peptide calibrators essential for reference measurement systems in laboratory medicine and quality control of pharmaceutical products [24]. The increasing use of peptides synthesised through solid phase peptide synthesis necessitates reliable methods for accurately assessing their purity. Various approaches, such as mass balance, amino acid analysis, qNMR, and nitrogen determination, can be employed to accurately determine the purity of peptide calibrators. However, it is crucial to account for structurally related peptide impurities to avoid any biases in purity assessment. Liquid chromatography coupled with high-resolution mass spectrometry (LC-hrMS) has emerged as a key technique for identifying and quantifying structurally related peptide impurities in intact peptide calibrator materials [24]. In this study, LC-hrMS-based methods were developed and validated in-house to accurately identify and quantify structurally related peptide impurities in synthetic Aβ1-40 and Aβ1-42 materials. Fig. 1 presents a summarized schematic diagram of the PICAA strategy used for preparing the primary standard for Aβ 1–40 and 1–42 peptides. Two separation methods were developed and used to separate and quantify potential co-eluting impurities. The first method utilised the Aeris Peptide C18 column (250 × 2 mm i.d., 5 µm particle size), and the second method used a ProSwiftTM RP-4H column (250 × 1.0 mm i.d., PS-DVB) from Thermo Scientific. In preliminary studies, the Aeris Peptide C18 column was found to be the most suitable for impurity separation. To analyze a standard mixture of Aβ1-40 and Aβ1-42 peptides, an optimised method using the Aeris Peptide C18 column was employed. The optimized method achieved complete separation of peptide-related impurities from the amyloid peptides. To detect trace peptide impurities, a sample of amyloid with a high concentration (approximately 200 μg/g) was used. To avoid contamination of the ionization source, the main component peak was cut off using a switch valve and directed to waste. Peak intensities higher than 10,000 cps in the full mass spectrum were carefully examined for impurities. Those, like the sodiated species and adducts of peptide and water, which are artificially generated during the ionization process, were excluded from the analysis.

This method helped identify two impurities for each peptide. These impurities were identified as shorter versions of the relevant peptide. The impurities likely eluted before the amyloid peak due to their comparatively lower hydrophobicity, which could be a result of incomplete synthesis or early termination of the peptide. Overall, the developed methods successfully separated and quantified impurities associated with the Aβ1-40 and Aβ1-42 peptides, revealing the presence of shorter peptide variants as impurities. During the peptide synthesis process, amino acid deletions or insertions, which involve the absence or addition of one or more amino acid residues, could occur. These modifications are commonly observed as impurities. Additionally, truncated peptide impurities, arising from the degradation of desired amino acid residues at either the N- or C-terminus during transportation or storage, are also categorized as amino acid deletion impurities. Table 2, Table 3 present the mass fractions of individual structurally related peptide impurities found in the Aβ1-40 and Aβ1-42 materials, respectively. These impurities contribute to a total mass fraction of 6.60 mg/g and 2.78 mg/g for the Aβ1-40 and Aβ1-42 materials, respectively. To determine the uncertainty associated with the total mass fraction, the expanded uncertainty was calculated at a 95 % confidence level, with a coverage factor (k) of 2. The resulting uncertainties are ± 0.22 mg/g and ± 0.08 mg/g for the Aβ1-40 and Aβ1-42 materials, respectively. These findings underscore the presence and quantification of various structurally related peptide impurities, including those resulting from truncated peptides, in the amyloid material.

**Table 2:** Amyloid β 1–40 identified peptide related impurities.

**Entity**	**Amino Acid Sequence**	**Parent Mass (/*m*/*z*)**	**Charged State**
**Aβ_1-40_**	D A E F R H D S G Y E V H H Q K L V F F A E D V G S N K G A I I G L M V G G V V	1083.29	+4
**Variant 1**	F A E D V G S N K G A I I G L M V G G V V	1017.54	+2
**Variant 2**	H D S G Y E V H H Q K L V F F A E D V G S N K G A I I G L M V G G V V	1238.29	+3

**Table 3:** Amyloid β 1–42 identified peptide related impurities.

**Entity**	**Amino Acid Sequence**	**Parent Mass (/*m*/*z*)**	**Charged State**
**Aβ_1-42_**	D A E F R H D S G Y E V H H Q K L V F F A E D V G S N K G A I I G L M V G G V V I A	1129.57	+4
**Variant 1**	D A E F R H D S G Y E V H H Q K L V F F A E D V G S N K G A I I G L M V G G	1133.32	+4
**Variant 2**	H D S G Y E V H H Q K L V F F A E D V G S N K G A I I G L M V G G V V I A	1299.33	+3

Table 4 provides a comprehensive summary of the purity information obtained for Aβ 1–40 and Aβ 1–42 using the PICAA strategy. The successful determination of their purity now allows these peptides to serve as primary calibrators for subsequent SPE/ID-LC/MSMS analysis, enabling accurate quantification of amyloid peptides in CSF. The implementation of the PICAA strategy, coupled with the development of validated LC/MS-based methods, allows for the reliable quantification of amyloid peptides in CSF. The purity information obtained for Aβ 1–40 and β 1–42 peptides using this approach enables their use as primary calibrators, providing a robust foundation for accurate and precise analysis in the field of amyloid peptide quantification.

**Table 4:** Aβ 1–40 and Aβ 1–42 purity information.

	**Aβ 1**–**40**	**Aβ 1**–**42**
**Source**	R-Peptide	R-Peptide
**Purity value (mg/g)**	517.1	440.44
**Expanded uncertainty of purity value (mg/g)**	72.8	27.1
**Traceability of purity value (Amino Acids)**	NMIJ (Amino acid CRMs) [25]	NMIJ (Amino acid CRMs) [25]
**Purity assay conducted**	PICAA	PICAA

### 3.2 SPE/ID-LC/MSMS

Interest in quantifying peptides and proteins to gain insights into biological processes during disease and therapeutic programs has steadily increased in recent years. Traditional methods for quantifying Aβ peptides in biological fluids, such as Western blots and ELISA, have limitations in terms of dynamic range, matrix interference, and assay linearity [26]. To address these challenges, we developed a high-throughput and cost-effective assay to accurately and reproducibly quantify Aβ1-42 and Aβ1-40 peptides in CSF. The method we developed utilized isotope dilution mass spectrometry (IDMS) for absolute quantification. For the experiments, we used a Q-Exactive High Resolution (HR) MS coupled with Dionex Ultimate 3000 UPLC. To enhance selectivity and separate the Aβ peptides from other high-abundance peptides in complex CSF samples, we performed SPE using Oasis MCX, which also provided clean-up. Measurement of Aβ42 in CSF is challenging due to its presence at low concentrations (pg/mL range), its poor aqueous solubility, non-specific binding to other peptides/proteins and/or to the walls of tubes and pipette tips, and a tendency to aggregate [8], [27]. The Oasis MCX sorbent is a novel, mixed-mode polymeric sorbent that has been optimized to achieve higher selectivity and sensitivity for extracting basic compounds with cation exchange groups, without requiring evaporation or reconstitution that can cause the loss of amyloid peptides [26].

**Fig. 2:**
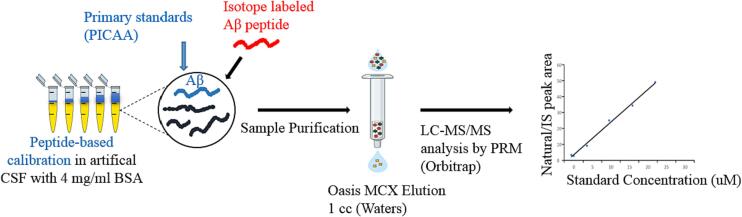
Workflow for Quantification of β-Amyloid peptides 1–40 and 1–42 in Artificial CSF by LC-MS/MS.

**Fig. 3:**
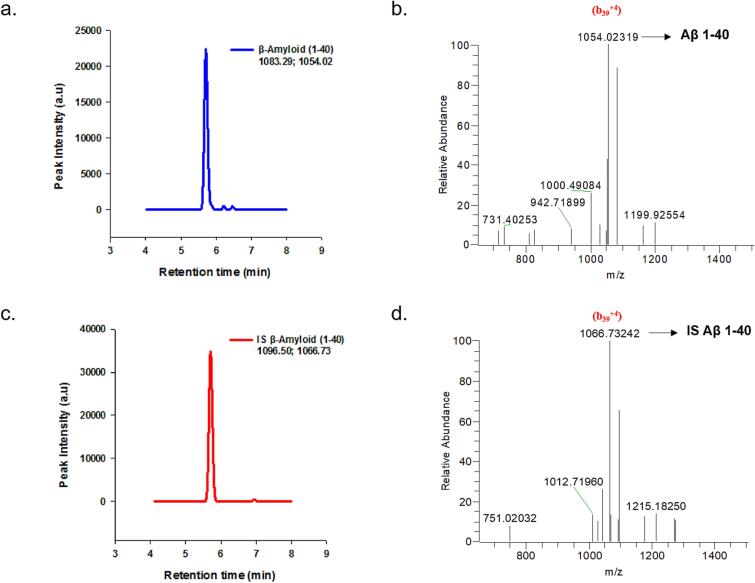
Positive ESI mass spectra of the Aβ1–40 peptides a) Total Ion Chromatogram (TIC) of Aβ1–40. b) Mass spectrum of Aβ1–40. c) TIC chromatogram of [15N] Aβ1–40. d) Mass spectrum of [15N] Aβ1–40.

**Fig. 4:**
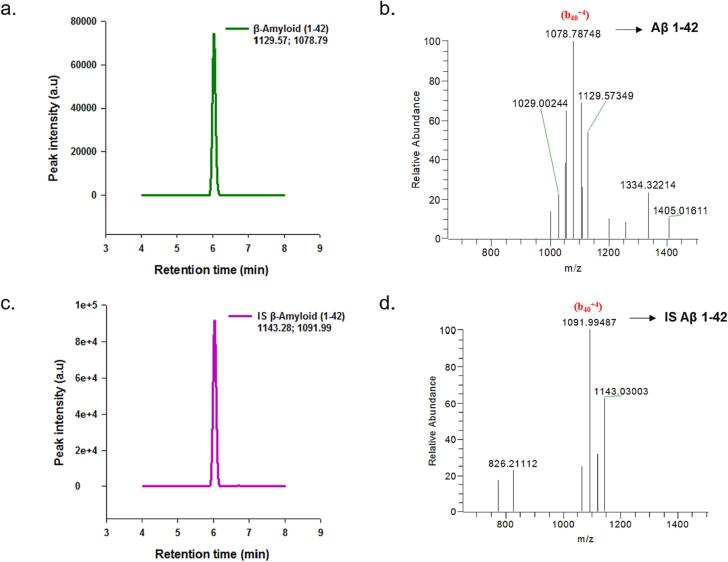
Positive ESI mass spectra of the Aβ1–42 peptides Total Ion Chromatogram (TIC) of Aβ1–42. b) Mass spectrum of Aβ1–42. c) TIC chromatogram of [15N] Aβ1–42. d) Mass spectrum of [15N] Aβ1–42.

Artificial CSF containing 4 mg/ml BSA served as a surrogate matrix. The use of a surrogate matrix is a common practice in analytical methods when the availability of the actual sample matrix is limited or problematic. Magdalena Korecka et al. established an artificial CSF matrix that contains 4 mg/mL of bovine serum albumin (BSA) as a surrogate matrix for calibration purposes [20]. Incorporating BSA into the artificial CSF matrix aims to mimic the matrix effects observed in real CSF samples, thereby allowing for more accurate and reliable calibration of the analytical method. The surrogate matrix, in this case, has demonstrated linearity and reproducibility comparable to using human pooled CSF as the calibration matrix. Fig. 2 illustrates the workflow for quantification of Aβ 1–40 and Aβ 1–42 in artificial CSF by SPE/ID-LC/MSMS. Analysis of the peptides was performed using electrospray ionization in the positive ion mode, where the peptides were detected as protonated species. Specifically, for the amyloid peptides, positive mode MS exhibited strong signals for the quadruply charged ions (Aβ 1–40 *m*/*z* 1083.29 and Aβ 1–42 *m*/*z* 1129.57), while the triply and doubly charged species showed comparatively weaker signals. To target specific ions for quantification, the (M − 4H)^4+^ ions of the amyloid peptides (Aβ 1–40 and Aβ 1–42) were selected for parallel reaction monitoring (PRM) transitions. Fig. 3 displays the total ion current (TIC) chromatograms and mass spectra of Aβ1–40 and [15N] Aβ1–40. Similarly, Fig. 4 presents the TIC chromatograms and mass spectra of Aβ1–42 and [15N] Aβ1–42. Table 1 provides the mass-to-charge ratio (*m*/*z*) of the amyloid peptides, along with their PRM transitions and optimized collision energies.

A six-point calibration was conducted using the PICAA-analyzed peptides spiked into a surrogate matrix as calibration material. This process led to the development of linear and reproducible calibration curves for both Aβ1-42 and Aβ1-40 peptides. To ensure clinical relevance, the calibration ranges for Aβ1-42 and Aβ1-40 peptides were established as 300–20000 pg/ml for both analytes. These ranges were chosen to encompass the expected concentrations of these peptides in clinical samples, ensuring that the method is capable of accurately quantifying the analytes at biologically relevant levels. The correlation coefficient (r) for the calibration curves was 0.999 for Aβ1-40 and 0.998 for Aβ1-42, indicating a strong relationship between the analyte concentration and response. The limits of detection (LOD) and limits of quantification (LOQ) were calculated according to the International Council for Harmonisation (ICH) defined criteria [28], [29]. The LOD, calculated as three times the standard deviation of the intercept divided by the slope of the calibration curve, was determined to be 100 pg/ml for Aβ1-40 and 140 pg/ml for Aβ1-42. The LOQ, calculated as ten times the standard deviation of the intercept divided by the slope of the calibration curve, was found to be 300 pg/ml for Aβ1-40 and 420 pg/ml for Aβ1-42. Table 5 summarizes the validation parameters obtained for the developed method, including the calibration range, correlation coefficient, LOD, and LOQ. These parameters provide important information on the sensitivity, linearity, and reliability of the method for quantifying Aβ1-40 and Aβ1-42 peptides.

**Table 5:** Linear range of measurement for quantification of Aβ1–40 and Aβ1–42 peptides.

**Parameters**	**Aβ1–40**	**Aβ1–42**
Linearity (pg/ml)	300–20000	300–20000
Correlation Coefficient	0.999	0.998
Limit of detection, LOD (pg/ml)	100	140
Limit of quantification, LOQ (pg/ml)	300	420

In terms of clinical relevance, the reported levels of Aβ1-40 in biological samples typically range from about 2–20 ng/mL, while Aβ1-42 levels are lower, ranging from 0.2 to 1 ng/mL [30], [31]. The sensitivity and detection limits achieved by the developed method fall within the range necessary for accurately quantifying these peptides in clinical samples. Overall, the validated method demonstrates good sensitivity, linearity and quantification capabilities for Aβ1-40 and Aβ1-42 peptides. This is particularly important considering the different levels of these peptides observed in clinical samples, where Aβ1-40 is typically present at higher concentrations compared to Aβ1-42. The ability of the method to accurately measure these peptides within the reported concentration ranges enables reliable analysis and monitoring of Aβ peptide levels, contributing to the understanding and diagnosis of various neurodegenerative diseases, including Alzheimer's disease.

The validity of the measurements in this study was confirmed through the analysis of quality control (QC) samples, which included a surrogate matrix spiked with primary calibration materials. According to the ICH Topic Q 2 (R1) Guide titled “Validation of Analytical Procedures: Text and Methodology”, accuracy is a parameter that evaluates the precision and trueness of test results [28]. To assess the precision of the measurement system, the method's repeatability and intermediate precision were evaluated. Repeatability was determined by analyzing QC solutions in replicates of four within the same day, while intermediate precision was determined by analyzing replicates of four samples on three different days. Table 6 summarizes the percent recovery and precision values for the Aβ1-40 and Aβ1-42 peptides obtained from the QC samples. In addition to assessing QC performance, the method was also employed to measure a pooled CSF sample from healthy donors. The results align well with the clinical levels in CSF reported for the healthy group, as shown in Table 6.

**Table 6:** Amyloid β % recovery and quantitative results.

**Peptide**		**Expected Concentration (ng/mL)**	**Measured Concentration (n = 4)(ng/mL)**	**Combined Uncertainty U (ng/ml) k = 2**	**Precision (%CV)**	**Recovery (%)**
**aβ 1**–**40**	QC-1	2.46	2.64	0.32	6.65	109.58
	QC-2	6.93	6.97	0.61	2.66	100.61
	Pooled CSF		4.95	0.47	3.35	
**aβ 1**–**42**	QC-1	2.03	2.08	0.35	7.98	102.40
	QC-2	5.84	5.26	0.57	6.74	90.03
	Pooled CSF		0.70	0,12	7.63	

A recovery above 90 % obtained for the QC samples suggests that the developed method effectively captures and quantifies the target analytes. This indicates that the extraction, sample preparation, and analytical steps involved in the procedure are robust and result in minimal losses or biases during the process. Precision, as indicated by the coefficient of variation (%CV), is an important measure of the repeatability and reliability of a measurement method. In this study, precision was assessed by calculating the %CV for the measured values of the analytes. Precision %CV of less than 10 % is generally considered excellent and indicates a high level of consistency and reproducibility in the measurements. A low %CV suggests that the method has minimal random errors and that the results obtained from repeated analyses are closely clustered around the mean value. These findings strengthen the validity and utility of the method for quantitative analysis in related research and clinical applications.

### 3.3 Uncertainty evaluation

The uncertainty evaluations for the SPE/ID-LC/MSMS analysis in this study followed the guidelines provided in the EURACHEM/CITAC Guide for Quantifying Uncertainty in Analytical Measurement [33] and The Fitness for Purpose of Analytical Method [32]. The current method considers several sources of uncertainty, including defined parameters such as the repeatability standard deviation (s_r_) (2), the contribution to total variation from the grouping factor (s_between_) (3), the uncertainty associated with the calibration curve (4), the uncertainty arising from the purity of peptides and the accuracy of the balance. The standard combined uncertainty is calculated using the provided formula (5). To achieve a level of confidence of approximately 95 %, the expanded uncertainty is determined by applying a coverage factor of 2. Table 7 presents the total peptide concentrations, along with the contributions of relative uncertainty for the Aβ1-40 peptide. Similarly, Table 8 provides the total peptide concentrations and relative uncertainty contributions for the Aβ1-42 peptide.

(2) $$s_{r}=\sqrt{MS_{\mathrm{withingroup}}}$$

(3) $$s_{\mathrm{between}}=\sqrt{\frac{MS_{\mathrm{within}}-MS_{\mathrm{between}}}{n_{\mathrm{repeat}}}}$$

(4) $$u_{\mathrm{calibration}}=\frac{s}{m}x\sqrt{\frac{1}{n_{\mathrm{rep}}}+\frac{1}{n_{\mathrm{cal}}}+\frac{{\left(x_{\mathrm{pred}}-x_{\mathrm{mean}}\right)}^{2}}{\sum {\left(x_{i}-x_{\mathrm{mean}}\right)}^{2}}}$$

(5) $$u_{\mathrm{combined}}=k\sqrt{\frac{u_{\mathrm{sr}}^{2}}{n_{\mathrm{rep}}}+\frac{u_{\mathrm{sbetween}}^{2}}{n_{\mathrm{days}}}+u_{\mathrm{calibration}}^{2}+u_{\mathrm{peptidepurity}}^{2}+u_{\mathrm{balance}}^{2}}$$

**Table 7:** Uncertainty budget β-amyloid 1–40.

	**Level1 (pg/ml)**	**Level2 (pg/ml)**	**Pooled CSF**
Mass Fraction	2641.72	6972.77	4947.79
Components (relative, %)			
S_between_	0.03	0.01	0.01
S_r_	0.03	0.02	0.02
Calibration Curve	0.002	0.001	0.001
Purity of Aβ1-40 peptide	0.04	0.04	0.04
The accuracy of Balance	8.08E-06	8.08E-06	8.08E-06
Combined Standard Uncertainty (pg/ml)	154.09	299.00	234.08
Expanded Standard Uncertainty	308.18	598.01	468.16

**Table 8:** Uncertainty budget β-amyloid 1–42.

	**Level1 (pg/ml)**	**Level2 (pg/ml)**	**Pooled CSF**
Mass Fraction	2080.43	5259.00	701.26
Components (relative, %)			
S_between_	0.02	0.04	0.01
S_r_	0.08	0.03	0.07
Calibration Curve	0.01	0.01	0.03
Purity of Aβ1-42 peptide	0.02	0.02	0.02
The accuracy of Balance	8.08E-06	8.08E-06	8.08E-06
Combined Standard Uncertainty (pg/ml)	164.87	268.14	59.62
Expanded Standard Uncertainty	329.74	536.30	119.25
			

Recently, a collaborative effort by the CSF biomarker working group, supported by the International Federation of Clinical Chemistry and the Institute for Reference Materials and Measurements, resulted in the preparation of the first certified reference material (CRM) for amyloid β1-42 [34]. This CRM serves the essential purpose of recalibrating commercial immunoassays and has been produced in three different levels: ERM-DA480/IFCC, ERM-DA481/IFCC, and ERM-DA482/IFCC [34]. The characterization of these CRMs was accomplished using isotope dilution mass spectrometry methods [34]. In our work, we also comprehensively characterized both Aβ1-40 and Aβ1-42 peptides using the PICAA approach. The ultimate goal of this project is to develop a CSF-based standard reference material for Aβ1-40 and Aβ1-42 peptides.

## 4 Conclusion

In conclusion, the accurate quantification of Aβ peptides in surrogate CSF is crucial for understanding their role as preclinical biomarkers in AD. This study successfully developed a method using a SPE and ID-LC/MSMS platform designed to simultaneously quantify Aβ 1–40 and Aβ 1–42 peptides in surrogate CSF. By effectively eliminating matrix interferences and utilizing internal standards ([15N] Aβ1-40 and [15N] Aβ1-42), this platform ensures accuracy and specificity in the quantification process. The comprehensive characterization of both Aβ1-40 and Aβ1-42 peptides using the PICAA approach was a crucial aspect of this study. The successful determination of the purity of these peptides using the PICAA strategy enabled their use as primary calibrators in subsequent SPE/ID-LC/MSMS analyses. This achievement is an initial step towards the ultimate goal of creating a CSF-based standard reference material for Aβ1-40 and Aβ1-42 peptides. The findings from this study provide a valuable tool for researchers studying Aβ peptides as preclinical biomarkers in Alzheimer's disease. The enhanced specificity and flexibility of the developed platform also have potential implications for AD diagnosis and future investigations of novel Aβ peptide biomarkers.

## Funding

This project European ReMiND project (nr:15HLT02) has received funding from the EMPIR programme co-financed by the Participating States and from the European Union’s Horizon 2020 research and innovation programme.

## Declaration of competing interest

The authors declare that they have no known competing financial interests or personal relationships that could have appeared to influence the work reported in this paper.
